# Newborn readmissions and virtual primary care delivery: a population-based case-control study

**DOI:** 10.1186/s12875-024-02478-2

**Published:** 2024-06-24

**Authors:** Eyal Cohen, Therese A. Stukel, Xuesong Wang, Azmina Altaf, Monica Kopec, Ori Davidov, Tharani Raveendran, Natasha R. Saunders

**Affiliations:** 1 The Hospital for Sick Children, 555 University Avenue, Toronto, ON M5G 1X8 Canada; 2 Department of Pediatrics, University of Toronto, Toronto, Canada; 3 ICES, Toronto, Canada; 4 Child Health Evaluative Sciences, SickKids Research Institute, Toronto, Canada; 5 Institute of Health Policy, Management and Evaluation, University of Toronto, Toronto, Canada; 6 Edwin S.H. Leong Centre for Healthy Children, University of Toronto, Toronto, Canada; 7 Department of Statistics, University of Haifa, Haifa, Israel

**Keywords:** Newborn, Infant, Readmission, Emergency department, Hospitalization, Virtual, In-person, Primary care, Pediatrics

## Abstract

**Background:**

Early post-discharge assessments for newborns are recommended. Virtual care has become more prevalent during the pandemic, providing an opportunity to better understand its impact on the quality of post-discharge newborn care. The objective of this study was to understand whether primary care visit modality (in-person vs. virtual) is associated with early newborn hospital readmissions and emergency department (ED) visits.

**Methods:**

We conducted a population-based, case-control study using linked health administrative databases between September 1, 2020 and March 31, 2022 in Ontario, Canada. We compared the modality of primary care visits among cases (hospital readmission within 14 days of life) and controls (newborns without a readmission), matched on infant sex, gestational age, and maternal parity. We included an alternative definition of cases as a composite of either a newborn hospital readmission or emergency department (ED) visit or in-hospital death within the first 14 days of life. Conditional logistic regression models were used to model odds ratios (ORs), comparing those exposed to a virtual visit versus in-person visit, adjusting for infant birth weight, birth hospitalization length of stay, neighbourhood level material deprivation, rurality and presence of active maternal comorbidities.

**Results:**

Among 73,324 eligible newborns, 2,220 experienced a hospital readmission within 14 days of life and were matched to 8,880 controls. Jaundice was the primary reason for readmission (75% of readmissions). Compared to newborns who were seen in-person post-discharge, newborns who were seen virtually had higher odds of hospital readmission (adjusted odds ratio [aOR] 1.41 (95% CI 1.09, 1.83); the magnitude of effect was not different using the composite outcome (aOR 1.35, 95% CI 1.05, 1.75).

**Conclusions:**

Newborns who receive a virtual post-discharge visit are more likely than those who receive an in-person visit to require hospital readmission.

**Supplementary Information:**

The online version contains supplementary material available at 10.1186/s12875-024-02478-2.

## Introduction

The American Academy of Pediatrics recommends that newborns receive an assessment by a health care provider within three to five days after birth and within 48 to 72 h after hospital discharge; for newborns delivered by caesarian section and whose hospital stay is 96 h or longer, this visit is expected within a week [1]. Such recommendations are based on known health risks to newborns in the first week of life, such as jaundice and feeding problems, that put newborns at risk of hospital readmission [2, 3]. Early primary care visits conducted within a few days of birth hospitalization discharge have been reported to reduce rehospitalization risk in some [4, 5], but not all [6, 7] studies. Such visits are typically conducted in an ambulatory setting by a physician or nurse [1]. These visits provide an opportunity for assessment of jaundice, feeding difficulties, hydration problems, suspected sepsis, and detection of congenital malformations not apparent on the initial examination, and facilitate provision of anticipatory guidance to caregivers including nutrition support, illness prevention, and infant safety [1].

At the onset of the COVID-19 pandemic, a rapid switch to virtual care occurred to replace in-person visits. For instance, during the first 9 months of the pandemic (March 1, 2020 – November 28, 2020), 53% of pediatric primary care visits were delivered virtually in Ontario [8]. Over time, although total virtual care provision has decreased, virtual visits remain an important modality of care for many clinical encounters with 40 to 80% of primary care visits across all age groups performed virtually [9]. It has been hypothesized that increased virtual care provision may lead to increased emergency department (ED) visits, as some clinical issues cannot be meaningfully addressed virtually (e.g., those requiring physical examination). However, this conjecture has not borne out in studies conducted to date [10].

The potential risk of virtual care, specifically as it relates to post-discharge visits for newborn infants, is important to understand as the common reasons for hospital readmissions are potentially preventable with early interventions resulting from in-person care (e.g. via a weight check; an observation of, and support for, feeding; and, a physical examination for jaundice). The objective of this study was to understand whether primary care visit modality (in-person vs. virtual) was associated with newborn hospital readmissions and ED visits. We hypothesized that newborns who have virtual post-discharge visits will experience higher rates of hospital readmission or ED visits compared with those who have in-person post discharge visits.

## Methods

### Study design and population

The study was conducted in Ontario, Canada’s largest province, with a population of 14.2 million as of 2021 [11], and approximately 140,000 births per year [12]. We conducted a population-based, case-control study using data obtained from linked health administrative databases available at ICES (formerly the Institute for Clinical Evaluative Sciences), an independent, non-profit research institute whose legal status under Ontario’s health information privacy law allows it to collect and analyze health care and demographic data, without consent, for health system evaluation and improvement. We included all term (≥ 37 weeks’ gestational age) singleton live infants born in hospital between September 1, 2020 and March 31, 2022 who experienced a single primary care visit within the first 7 days of life and were eligible for Ontario’s universal provincial health insurance [13]. In Ontario, all Canadian Citizens, permanent residents, or holders of work permits with a primary residence in Ontario are eligible for provincial health insurance, as are their newborns. Newborns with multiple visits during the exposure period were excluded. March 1, 2020 to August 31, 2020 was considered a wash-out period and was excluded due to unstable fluctuations in health care usage and lack of in-person care visit availability during the early pandemic period. We excluded infants born with higher anticipated health care needs, including those with complex chronic conditions identified during the birth hospitalization or within 7 days of birth, and those with prolonged birth hospitalizations (hospital discharge > 48 h for vaginal deliveries and > 72 h for C-section) [14]. We also excluded infants with likely implausible gestational age (> 42 weeks) and term birth weights (< 1 kg or > 6 kg). Lastly, we excluded infants with incomplete or invalid data (birth dates, death dates, sex, gestational age) and invalid or incomplete maternal parity data.

### Outcomes

The first date of the eligibility for the outcome (index date) was the date of discharge from the birth hospitalization. Cases were defined as having the primary outcome of a hospital readmission within 14 days of life. Controls were derived from those newborns who did not experience a hospital readmission within 14 days of life. We included an alternative definition of the primary outcome as a composite of either a newborn hospital readmission or ED visit or in-hospital death within the first 14 days of life. For this composite outcome, controls were defined as newborns with no readmission to a hospital and/or ED visit within 14 days of life.

### Exposure

We categorized all infants with a single visit to a primary care provider within 7 days of life, into those with an in-person visit and those with a virtual visit. Visits that occurred on the date of readmission were excluded.

### Data sources and variables

The ICES MOMBABY database was used to ascertain information about birth mothers and their newborn infants via linked mother-infant birth hospitalization records [15]. This dataset identifies discharges for obstetrical deliveries ≥ 20 weeks gestation, links maternal and newborn Canadian Institute for Health Information (CIHI)-Discharge Abstract Database data and holds records for 98% of Ontario births. The Ontario Health Insurance Plan (OHIP) physician billings database was used to ascertain primary care visit modality using in-person and virtual codes [16]. ED visits were obtained from the CIHI-National Ambulatory Care Reporting System (NACRS) [17], and hospitalization data was obtained from the CIHI-Discharge Abstract Database [18]. We obtained demographic information (date of birth, sex, and postal code) from Ontario’s Registered Persons Database [19]. Individual postal codes were used to ascertain the Ontario Marginalization Index (ON-Marg) based on data from the 2016 Canadian Census [20] and were linked to the Statistics Canada Postal Code Conversion File (PCCF) to determine rural residence (community size ≤ 10,000) [21]. ON-Marg outlines neighbourhood material deprivation, combining census information on income and education calculated at the dissemination area level (approximately 400 to 700 persons) and was used as a measure of socioeconomic status [20]. Quintiles are used to define the marginalization index, with 1 representing the least deprived neighbourhoods and 5 representing the most deprived neighbourhoods [20]. Maternal immigration data (refugee immigrant, non-refugee immigrant, non-immigrant), was obtained from Immigration, Refugees and Citizenship Canada’s (IRCC) Permanent Resident Database [22]. Maternal comorbidities were ascertained from a 2-year look-back period from the inftant’s date of birth, and included gestational diabetes, pre-pregnancy diabetes [from the Ontario Diabetes Dataset], chronic hypertension, pre-eclampsia and active severe mental illness (defined as any hospitalization or ED visit for mental illness) [23]. Diagnostic codes for ascertainment of these comorbidities are summarized in Supplementary eTable 3. These datasets were linked using unique encoded identifiers and analyzed at ICES.

### Statistical analyses

Each case was matched to 4 controls (without replacement) on sex assigned at birth of the infant (male or female), gestational age of the infant at delivery (37, 38, 39, 40, 41 or 42 weeks) and maternal parity (0, 1, or ≥ 2 births). We report the baseline characteristics of newborns and their mothers with balance between groups ascertained using standardized differences, using a standardized difference of < 0.1 as indicative of good balance between groups for a given characteristic [24]. We report the most responsible reason for newborn readmission among cases. Separate conditional logistic regression models were used to model odds ratios (ORs) for the primary outcome and the primary composite outcome, comparing those exposed to a virtual visit with those exposed to an in-person visit (the referent) [25]. Each model was adjusted for birth weight of the baby, birth hospitalization length of stay (< 24 h or ≥24 h) material deprivation quintile, rurality and active maternal comorbidities. A sensitivity analysis using the same regression modeling technique was performed, extending the exposure period to allow for a visit to a primary care provider within 10 days of life to include families who may have had difficulty accessing newborn care during the recommended time window. We conducted statistical analyses using SAS software, version 9.4 (SAS Institute Inc.).

## Results

### Baseline characteristics of birth mothers and newborns

**Fig. 1:**
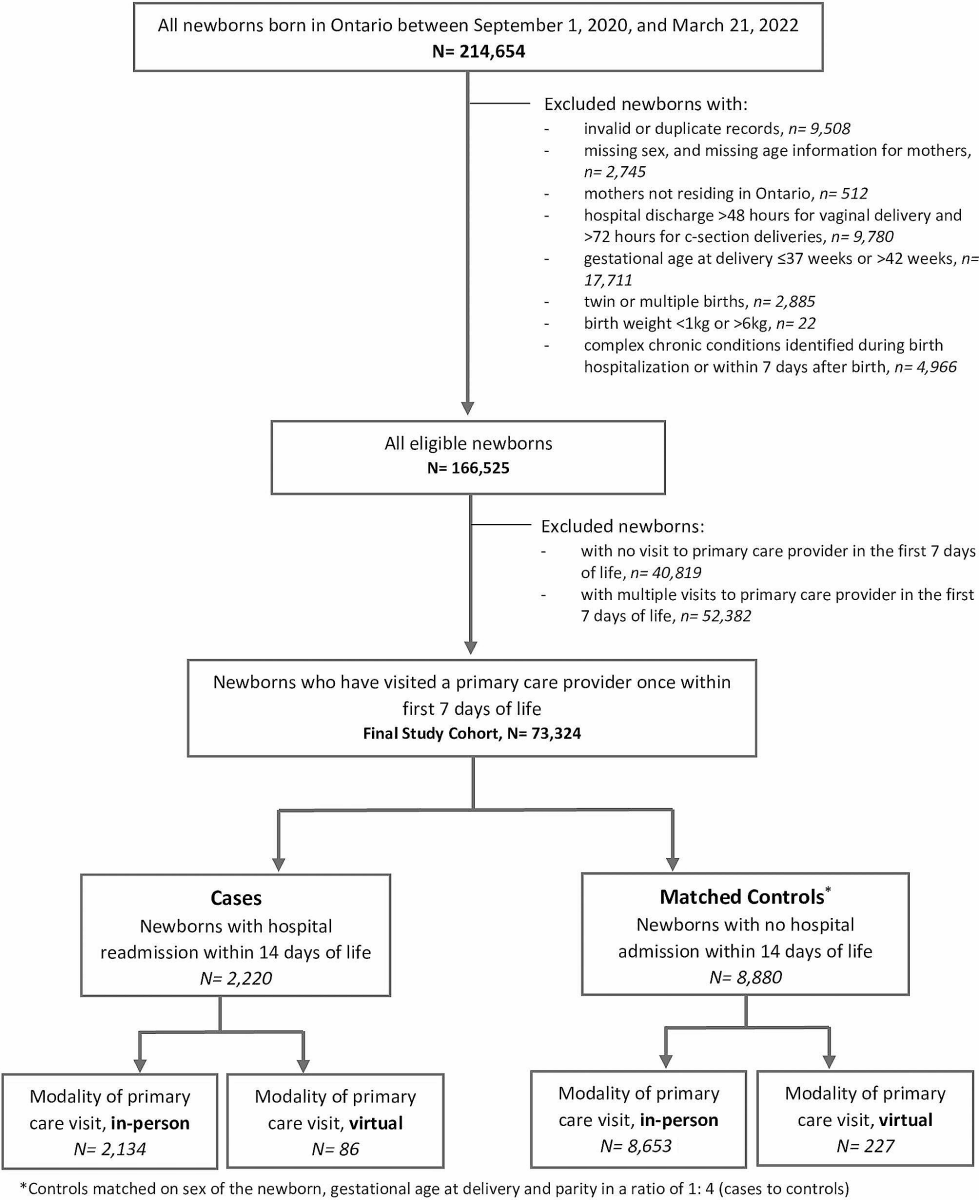
Study flow diagram of newborns born to mothers residing in Ontario between September 1, 2020, and March 31, 2022

Among 166,525 eligible newborns born during the study period, we excluded those with no primary care visits and those with multiple primary care visits in the first 7 days, leaving 73,324 newborns in the study (Fig. 1). Of those, 2,220 (3.0%) were identified as having a hospital readmission within 14 days of life (cases) and were matched to 8,880 controls (newborns who were not readmitted within 14 days of life) (Fig. 1). 55% of newborns were male and 51% were born to nulliparous mothers (Table 1). There were no differences in measured demographic characteristics of cases and controls. A greater proportion of cases (9.2%) had birth hospitalization stays < 24 h compared to controls (3.3%). A lower proportion of cases had mothers who were immigrants and refugees. Baseline characteristics of cases and controls in the sensitivity analysis were similar to those in the main analysis (Supplementary eTable 1).

**Table 1:** Baseline characteristics of mothers and newborns in Ontario between September 1, 2020 and March 31, 2022, who visited a primary care provider within 7 days of life, by primary outcome. All values represent n (%) unless otherwise indicated

		Before matching			Matched cohort		
		**Cases** Neonates with hospital readmission within 14 days of life	**Controls** Neonates with no hospital readmission within 14 days of life	Standardized difference	**Cases** Neonates with hospital readmission within 14 days of life	**Controls** Neonates with no hospital readmission within 14 days of life	Standardized difference
	Sample Size (N)	2,220	71,104		2,220	8,880	
**Matching Factors**							
Male	Yes	1,224 (55.1)	35,730 (50.3)	0.10	1,224 (55.1)	4,896 (55.1)	
Gestational Age in Weeks*	37	525 (23.6)	5,523 (7.8)	0.45	525 (23.6)	2,100 (23.6)	
	38	610 (27.5)	17,879 (25.1)	0.05	610 (27.5)	2,440 (27.5)	
	39	641 (28.9)	25,716 (36.2)	0.16	641 (28.9)	2,564 (28.9)	
	40	336 (15.1)	15,907 (22.4)	0.19	336 (15.1)	1,344 (15.1)	
	*41–42	108 (4.9)	6,079 (8.5)	0.15	108 (4.9)	432 (4.9)	
Parity	0	1,142 (51.4)	29,577 (41.6)	0.20	1,142 (51.4)	4,568 (51.4)	
	1	719 (32.4)	27,252 (38.3)	0.12	719 (32.4)	2,876 (32.4)	
	≥ 2	359 (16.2)	14,275 (20.1)	0.10	359 (16.2)	1,436 (16.2)	
**Demographic Factors**							
Material Deprivation Quintile	1 (Least Deprived)	434 (19.5)	15,817 (22.2)	0.07	434 (19.5)	1,928 (21.7)	0.05
	2	418 (18.8)	14,542 (20.5)	0.04	418 (18.8)	1,792 (20.2)	0.03
	3	469 (21.1)	13,503 (19.0)	0.05	469 (21.1)	1,717 (19.3)	0.05
	4	449 (20.2)	13,211 (18.6)	0.04	449 (20.2)	1,761 (19.8)	0.01
	5 (Most Deprived)	411 (18.5)	13,576 (19.1)	0.02	411 (18.5)	1,625 (18.3)	0.01
	Missing	39 (1.8)	455 (0.6)	0.10	39 (1.8)	57 (0.6)	0.10
Rurality*	Rural	237 (10.7)	5,765 (8.1)	0.09	237 (10.7)	717 (8.1)	0.09
	*Urban	1,983 (89.3)	65,339 (91.9)	0.09	1,983 (89.3)	8,163 (91.9)	0.09
**Neonatal Factors**							
Birth Weight	Mean (SD)	3364.0 (479.4)	3387.8 (449.8)	0.05	3364.0 (479.4)	3297.2 (469.6)	0.14
Birth Hospitalization Length of Stay < 24 h	Yes	205 (9.2)	2,919 (4.1)	0.21	205 (9.2)	294 (3.3)	0.25
**Maternal Factors**							
Age at Delivery	Mean (SD)	31.4 (5.1)	31.7 (4.9)	0.06	31.4 (5.1)	31.5 (5.1)	0.03
Maternal Comorbidity*	Gestational Diabetes	314 (14.1)	7,962 (11.2)	0.09	314 (14.1)	1,273 (14.3)	0.01
	Pre-existing Diabetes	112 (5.0)	2,395 (3.4)	0.08	112 (5.0)	415 (4.7)	0.02
	Hypertension	89 (4.0)	1,990 (2.8)	0.07	89 (4.0)	289 (3.3)	0.04
	Pre-eclampsia	1–5	82–86	0.02	1–5	14–18	0.00
	Active Severe Mental Illness	53 (2.4)	1,166 (1.6)	0.05	53 (2.4)	163 (1.8)	0.04
Immigration Status	Refugee	93 (4.2)	3,766 (5.3)	0.05	93 (4.2)	405 (4.6)	0.02
	Immigrant	525 (23.6)	19,762 (27.8)	0.10	525 (23.6)	2,562 (28.9)	0.12
	Non-Immigrant	1,602 (72.2)	47,576 (66.9)	0.11	1,602 (72.2)	5,913 (66.6)	0.12

*Small cells ≤ 5 have been suppressed and added to the largest category or expressed as ranges

**Table 2:** Baseline characteristics of mothers and newborns in Ontario between September 1, 2020 and March 31, 2022, who visited a primary care provider within 10 days of life, by primary outcome. All values represent n (%) unless otherwise indicated

		Before matching			Matched cohort		
		**Cases** Neonates with hospital readmission within 14 days of life	**Controls** Neonates with no hospital readmission within 14 days of life	Standardized difference	**Cases** Neonates with hospital readmission within 14 days of life	**Controls** Neonates with no hospital readmission within 14 days of life	Standardized difference
	Sample Size (N)	1,730	60,274		1,730	6,920	
**Matching Factors**							
Male	Yes	982 (56.8)	30,186 (50.1)	0.13	982 (56.8)	3,928 (56.8)	
Gestational Age in Weeks*	37	365 (21.1)	4,614 (7.7)	0.39	365 (21.1)	1,460 (21.1)	
	38	464 (26.8)	14,783 (24.5)	0.05	464 (26.8)	1,856 (26.8)	
	39	531 (30.7)	21,840 (36.2)	0.12	531 (30.7)	2,124 (30.7)	
	40	275 (15.9)	13,636 (22.6)	0.17	275 (15.9)	1,100 (15.9)	
	*41–42	95 (5.5)	5,401 (9.0)	0.13	95 (5.5)	380 (5.5)	
Parity	0	873 (50.5)	24,107 (40.0)	0.21	873 (50.5)	3,492 (50.5)	
	1	544 (31.4)	23,137 (38.4)	0.15	544 (31.4)	2,176 (31.4)	
	≥ 2	313 (18.1)	13,030 (21.6)	0.09	313 (18.1)	1,252 (18.1)	
**Demographic Factors**							
Material Deprivation Quintile	1 (Least Deprived)	319 (18.4)	12,624 (20.9)	0.06	319 (18.4)	1,402 (20.3)	0.05
	2	328 (19.0)	12,149 (20.2)	0.03	328 (19.0)	1,427 (20.6)	0.04
	3	363 (21.0)	11,479 (19.0)	0.05	363 (21.0)	1,322 (19.1)	0.05
	4	371 (21.4)	11,436 (19.0)	0.06	371 (21.4)	1,328 (19.2)	0.06
	5 (Most Deprived)	318 (18.4)	12,134 (20.1)	0.04	318 (18.4)	1,388 (20.1)	0.04
	Missing	31 (1.8)	452 (0.7)	0.09	31 (1.8)	53 (0.8)	0.09
Rurality*	Rural	236 (13.6)	5,999 (10.0)	0.12	236 (13.6)	669 (9.7)	0.12
	*Urban	1,494 (86.4)	54,275 (90.0)	0.11	1,494 (86.4)	6,251 (90.3)	0.12
**Newborn Factors**							
Birth Weight	Mean (SD)	3398.21 (491.81)	3398.11 (450.50)	0.00	3398.21 (491.81)	3312.72 (463.69)	0.18
Birth Hospitalization Length of Stay < 24 h	Yes	205 (11.8)	2,979 (4.9)	0.25	205 (11.8)	260 (3.8)	0.31
**Maternal Factors**							
Age at Delivery	Mean (SD)	31.08 (5.17)	31.46 (4.95)	0.08	31.08 (5.17)	31.27 (5.13)	0.04
Maternal Comorbidity*	Gestational Diabetes	246 (14.2)	6,556 (10.9)	0.10	246 (14.2)	902 (13.0)	0.04
	Pre-existing Diabetes	76 (4.4)	1,959 (3.3)	0.06	76 (4.4)	283 (4.1)	0.02
	Hypertension	66 (3.8)	1,690 (2.8)	0.06	66 (3.8)	244 (3.5)	0.02
	Pre-eclampsia	*1–5	*64–68	0.02	*1–5	*7–11	0.03
	Active Severe Mental Illness	43 (2.5)	1,091 (1.8)	0.05	43 (2.5)	149 (2.2)	0.02
Immigration Status	Refugee	67 (3.9)	3,274 (5.4)	0.07	67 (3.9)	336 (4.9)	0.05
	Immigrant	375 (21.7)	15,570 (25.8)	0.10	375 (21.7)	1,856 (26.8)	0.12
	Non-Immigrant	1,288 (74.5)	41,430 (68.7)	0.13	1,288 (74.5)	4,728 (68.3)	0.14

*Small cells ≤ 5 have been suppressed, and added to the largest category or expressed as ranges

### Main analysis

**Fig. 2:**
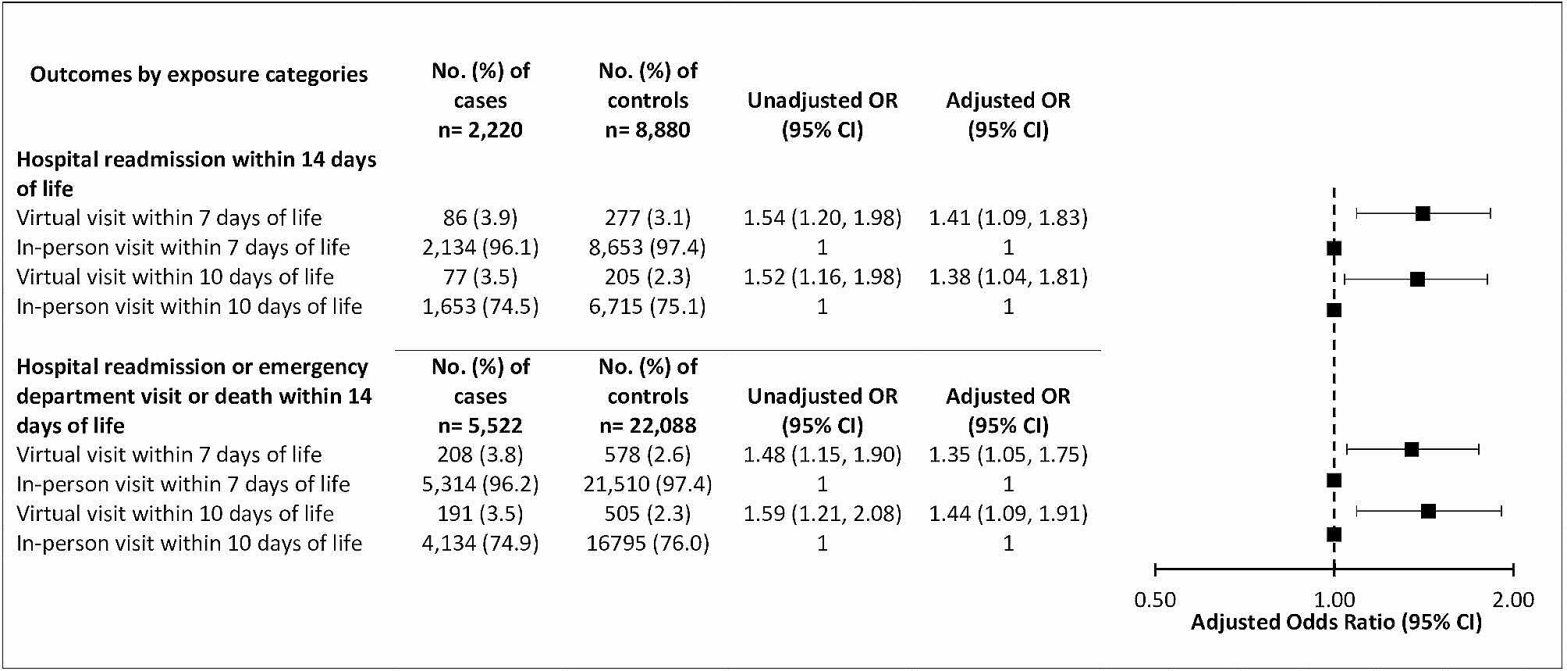
Forest plot depicting the primary outcome, newborns hospital readmissions within 14 days of life, and the composite outcome, hospital readmission, emergency department visit, or death within 14 days of life, for newborns in Ontario between September 1, 2020 and March 31, 2022. Odds ratios were adjusted for birth weight of the baby, maternal comorbidities, material deprivation quintile of the Ontario Marginalization Index, and rurality

Receipt of a virtual visit within 7 days of life was low in cases (3.9%) and controls (2.6%). Newborns who were hospitalized were more likely to receive a virtual visit (adjusted odds ratio [aOR] 1.41 (95% CI 1.09, 1.83) than those who were not readmitted; the magnitude of effect was similar using the composite outcome of hospital readmission, emergency department visit or death within 14 days of life (aOR 1.35, 95% CI 1.05, 1.75) (Fig. 2).

### Reason for readmission

Among cases, the most common reason for readmission in newborns was for jaundice (69.8% of readmissions in the virtual group, 71.6% in the in-person group) followed by infections (11.6% virtual group, 7.4% in-person group) and feeding problems (7.0% virtual group, 9.7% in-person group) (eTable 2).

### Sensitivity analyses

In extending the exposure period to allow for a primary care provider visit within 10 days of life, the adjusted odds of hospital readmission were higher in those who had a virtual visit compared to those who had an in-person visit (4.5% cases vs. 3.0% controls; aOR 1.38, 95% CI 1.04, 1.81) and for the composite outcome of readmission, ED visit, or death (4.4% vs. 2.9%, aOR 1.44, 95% CI 1.09, 1.91) (Fig. 2).

## Discussion

This population-based case-control study utilized a unique natural history experiment at a time during the COVID-19 pandemic when primary care providers in Ontario, Canada were being remunerated for both in-person and virtual post-discharge visits. We found that despite the availability of virtual care, most newborns during this time attended in-person post-discharge visits. Those newborns who had a virtual post-discharge visits experienced 41% higher odds of hospital readmission compared with those who had an in-person post discharge visit, and 35% increased odds of a composite outcome of readmission, ED visit or death. Jaundice was the predominant reason for readmission to hospital. Taken together, the findings from the study provide support for in-person post-discharge visits for healthy newborns.

The rapid shift during the pandemic to more virtual care has provided an opportunity to evaluate the efficacy and safety of this modality of care. Virtual care has been reported to be as effective as in-person care for a variety of pediatric conditions such as asthma [26] and abdominal pain [27], but, to our knowledge, our study is the first to report on severe outcomes related to the modality of post-discharge newborn care. Pandemic-specific factors may have contributed to our findings as the risk of newborn rehospitalization may have changed during this time. While some studies suggest that the frequency of overall post-discharge newborn rehospitalization did not change during the pandemic [28, 29], there have been reports of increased rehospitalization risk in some subgroups such as those born to nulliparous mothers [30] and those readmitted for hyperbilirubinemia [31]. One nation-wide study from England found that while acute care presentations decreased by 16.7% overall during vs. before the pandemic, attendance for feeding problems and neonatal jaundice increased by 7.5% and 12.8% respectively [32]. Such presentations may - in particular - rely on in-person primary care assessment to help prevent poor outcomes. While some aspects of feeding assessment can likely be conducted virtually for all babies (e.g., feeding history), others may require in-person care (e.g., obtaining an accurate weight, physical examination for dehydration such as assessment of the fontanelle or skin turgor), and others may be challenging in virtual settings (e.g., assessment for jaundice in suboptimal lighting and responsiveness to handling).

### Strengths and limitations

The newborns in the study were selected from a large, ethnically diverse population, limiting the risk of selection bias. However, the study has important limitations. First, while we were able to account for many important potential confounders including socio-demographic and some clinical characteristics, in our patient-level analysis, there is likely residual confounding from unmeasured factors such as breastfeeding, bilirubin levels at hospital discharge, supplementary supports (e.g., nurse or midwife home visits), race/ethnicity, provider and local hospital factors, access to laboratory services, and transportation. We cannot generalize our findings to scenarios where newborns had multiple post-discharge visits including those where some were virtual and some were in-person; such newborns may have widely varying patterns of virtual vs. in-person visits. They may also be at higher risk of rehospitalization as the underlying reason(s) for multiple visits may be due to factors that put them at risk for poor outcomes. Information on other important outcome measures such as satisfaction with care were not available in administrative databases. Our study was conducted in a jurisdiction with a single-payer universal healthcare system that provided remuneration for virtual visits during the study period; extrapolation of findings to other jurisdictions with different access to care may be limited. In Ontario, though virtual care utilization has waned from its peak, as of August 2023, 13% of primary care visits continued to be performed virtually, and the province’s single payer (the Ontario Ministry of Health) has introduced permanent fee codes for virtual care services provided by phone, video, text, and email, ensuring that patients – of any age - can access virtual care for any insured health care service that can be appropriately delivered through electronic means [33–35]. Thus, with future public health emergencies and/or with local circumstances during which virtual care may be a preferred option for families, providers, or health systems, evaluating trade-offs of virtual vs. in-person care in a variety of clinical contexts remains important.

## Conclusions

Newborns during the pandemic who had a virtual post-discharge visits experienced 41% higher odds of hospital readmission compared with those who had an in-person post discharge visit. These findings provide support for in-person post-discharge visits for healthy newborns. Given the widespread and rapid implementation of virtual care in recent years, ongoing monitoring and evaluation of the safety and effectiveness of virtual care and drivers of its use are paramount.

### Electronic supplementary material

Below is the link to the electronic supplementary material.

**eTable 1.** Baseline characteristics of mothers and newborns born in Ontario between September 1st, 2020 and March 31st, 2022 with a visit to a primary care provider within 7 days of life, by composite outcome. All values represent n (%) unless otherwise indicated.
** eTable 2.** Most responsible diagnosis group among cases (newborns who had at least one hospital readmission within 14 days).
** eTable 3.** List of complex chronic condition codes and corresponding ICD-10 Diagnosis and Procedure Codes.

## Data Availability

The data sets from this study are held securely in coded form at ICES. Data-sharing agreements prohibit ICES from making the data sets publicly available, but access may be granted to those who meet pre-specified criteria for confidential access, available at www.ices.on.ca/DAS. The complete data set creation plan, and underlying analytic code are available from the authors upon request, understanding that the programs may rely upon coding templates or macros unique to ICES.

## Abbreviations

aOR: adjusted odds ratio
ED: emergency department
IRCC: Immigration, Refugees and Citizenship Canada
OHIP: Ontario Health Insurance Plan
ON-Marg: Ontario Marginalization Index
OR: odds ratio
