# Supplementary material for: Newborn readmissions and virtual primary care delivery: a population-based case-control study
Source: BMC Prim Care. 2024 Jun 24;25:226. doi: 10.1186/s12875-024-02478-2 (PMC11194968; doi:10.1186/s12875-024-02478-2)
Supplement: Supplementary file 1 — eTable 1. Baseline characteristics of mothers and newborns born in Ontario between September 1st, 2020 and March 31st, 2022 with a visit to a primary care provider within 7 days of life, by composite outcome. All values represent n (%) unless otherwise indicated. eTable 2. Most responsible diagnosis group among cases (newborns who had at least one hospital readmission within 14 days). eTable 3. List of complex chronic condition codes and corresponding ICD-10 Diagnosis and Procedure Codes. [file 12875_2024_2478_MOESM1_ESM.docx]

**eTable 1.** Baseline characteristics of mothers and neonates born in Ontario between September 1st, 2020 and March 31st, 2022 with a visit to a primary care provider within 7 days of life, by composite outcome. All values represent n (%) unless otherwise indicated.

|  |  | **Before matching** | | | **Matched cohort** | | |
| --- | --- | --- | --- | --- | --- | --- | --- |
|  |  | **Cases** Neonates with hospital readmission or ED visit or death within 14 days of life | **Controls** Neonates with no hospital readmission within 14 days of life | Standardized difference | **Cases** Neonates with hospital readmission or ED visit or death within 14 days of life | **Controls** Neonates with no hospital readmission within 14 days of life | Standardized difference |
|  | Sample size (N) | 5,522 | 67,802 |  | 5,522 | 22,088 |  |
| **Matching Factors** | | | | | | | |
| Male | Yes | 2,998 (54.3) | 33,956 (50.1) | 0.08 | 2,998 (54.3) | 11,992 (54.3) |  |
| Gestational Age in Weeks* | 37 | 812 (14.7) | 5,236 (7.7) | 0.22 | 812 (14.7) | 3,248 (14.7) |  |
|  | 38 | 1,490 (27.0) | 16,999 (25.1) | 0.04 | 1,490 (27.0) | 5,960 (27.0) |  |
|  | 39 | 1,773 (32.1) | 24,584 (36.3) | 0.09 | 1,773 (32.1) | 7,092 (32.1) |  |
|  | 40 | 1,055 (19.1) | 15,188 (22.4) | 0.08 | 1,055 (19.1) | 4,220 (19.1) |  |
|  | *41 - 42 | 392 (7.1) | 5,795 (8.5) | 0.05 | 392 (7.1) | 1,568 (7.1) |  |
| Parity | 0 | 2,855 (51.7) | 27,864 (41.1) | 0.21 | 2,855 (51.7) | 11,420 (51.7) |  |
|  | 1 | 1,828 (33.1) | 26,143 (38.6) | 0.11 | 1,828 (33.1) | 7,312 (33.1) |  |
|  | ≥2 | 839 (15.2) | 13,795 (20.3) | 0.14 | 839 (15.2) | 3,356 (15.2) |  |
| **Demographic Factors** | | | | | | | |
| Material Deprivation Quintile | 1(Least Deprived) | 1,115 (20.2) | 15,136 (22.3) | 0.05 | 1,115 (20.2) | 4,941 (22.4) | 0.05 |
|  | 2 | 1,074 (19.4) | 13,886 (20.5) | 0.03 | 1,074 (19.4) | 4,474 (20.3) | 0.02 |
|  | 3 | 1,072 (19.4) | 12,900 (19.0) | 0.01 | 1,072 (19.4) | 4,245 (19.2) | 0.01 |
|  | 4 | 1,108 (20.1) | 12,552 (18.5) | 0.04 | 1,108 (20.1) | 4,156 (18.8) | 0.03 |
|  | 5(Most Deprived) | 1,082 (19.6) | 12,905 (19.0) | 0.01 | 1,082 (19.6) | 4,133 (18.7) | 0.02 |
|  | Missing | 71 (1.3) | 423 (0.6) | 0.07 | 71 (1.3) | 139 (0.6) | 0.07 |
| Rurality | Rural | 582 (10.5) | 5,420 (8.0) | 0.09 | 582 (10.5) | 1,756 (8.0) | 0.09 |
|  | Urban | 4,930 (89.3) | 62,277 (91.9) | 0.09 | 4,930 (89.3) | 20,301 (91.9) | 0.09 |
|  | Missing | 10 (0.2) | 105 (0.2) | 0.01 | 10 (0.2) | 31 (0.1) | 0.01 |
| **Neonatal Factors** | | | | | | | |
| Birth Weight | Mean (SD) | 3371.09 (458.84) | 3388.40 (450.04) | 0.04 | 3371.09 (458.84) | 3345.36 (462.81) | 0.06 |
| Birth Hospitalization Length of Stay <24 Hours | Yes | 329 (6.0) | 2,795 (4.1) | 0.08 | 329 (6.0) | 759 (3.4) | 0.12 |
| **Maternal Factors** | | | | | | | |
| Age at Delivery | Mean (SD) | 31.08 (5.06) | 31.71 (4.87) | 0.13 | 31.08 (5.06) | 31.50 (4.99) | 0.08 |
| Maternal Comorbidity | Gestational Diabetes | 694 (12.6) | 7,582 (11.2) | 0.04 | 694 (12.6) | 2,694 (12.2) | 0.01 |
|  | Pre-existing Diabetes | 236 (4.3) | 2,271 (3.3) | 0.05 | 236 (4.3) | 805 (3.6) | 0.03 |
|  | Hypertension | 197 (3.6) | 1,882 (2.8) | 0.05 | 197 (3.6) | 633 (2.9) | 0.04 |
|  | Pre-eclampsia | 7 (0.1) | 80 (0.1) | 0.00 | 7 (0.1) | 41 (0.2) | 0.02 |
|  | Active Severe Mental Illness | 148 (2.7) | 1,071 (1.6) | 0.08 | 148 (2.7) | 374 (1.7) | 0.07 |
| Immigration Status | Refugee | 263 (4.8) | 3,596 (5.3) | 0.03 | 263 (4.8) | 1,072 (4.9) | 0.00 |
|  | Immigrant | 1,431 (25.9) | 18,856 (27.8) | 0.04 | 1,431 (25.9) | 6,104 (27.6) | 0.04 |
|  | Non-Immigrant | 3,828 (69.3) | 45,350 (66.9) | 0.05 | 3,828 (69.3) | 14,912 (67.5) | 0.04 |

*Small cells ≤5 have been suppressed, and added to the largest category or expressed as ran

**eTable 2.** Most common responsible diagnosis group among cases (neonates who had at least one hospital readmission within 14 days). All values represent n (%).

|  | **Visit to Primary Health Care Provider Within 7 Days of Life (N=2,220)** | | |
| --- | --- | --- | --- |
| Reason for Hospital Readmission | Virtual  N=86 | In-person  N=2,134 | Total  N=2,220 |
| Jaundice | 60 (69.8) | 1,528 (71.6) | 1,588 (71.5) |
| Feeding Problems | 6 (7.0) | 208 (9.7) | 214 (9.6) |
| Infection | 10 (11.6) | 157 (7.4) | 167 (7.5) |
| Respiratory | 1 (1.2) – 5 (5.8) | 44 (2.1) | 45 (2.0) – 49 (2.2) |
| Other | 5 (5.8) – 9 (10.5) | 197 (9.2) | 202 (9.1) – 206 (9.3) |

**eTable 3.** List of complex chronic condition codes and corresponding ICD-10 Diagnosis and Procedure Codes.

| **Categories** | **Subcategories** | **ICD-10** |
| --- | --- | --- |
| **Neurologic and Neuromuscular** | Brain and spinal cord malformations | Q00-Q07, G90.1 |
|  | Mental retardation | F71-F73 |
|  | CNS degeneration and diseases | E75.0, E75.1, E75.2, E75.4, F84.2, G11.1-G11.4, G11.8, G11.9, G12.0- G12.2, G12.8, G12.9, G31.01, G31.09, G31.8, G31.89, G32.89, G93.8, G93.9, G94, G91.1, G31.9, G25.3, G95.19, G95.89, G90.9, Q85.1 |
|  | Infantile cerebral palsy | G80 |
|  | Epilepsy | G40.311, G40.301, G40.211, G40.219, G40.411, G40.419, G40.111, G40.119, G40.804, G40.911, G40.919 |
|  | Other disorders of CNS | G37.1, G37.2, G37.8, G81.90, G82.90, G82.50-G82.54, G83.5, G83.9, G93.1, G93.5, R40.3, 0016070, 0016071, 0016072, 0016073, 0016074, 0016075, 0016076, 0016077, 0016078, 001607B, 0016370, 0016371, 0016372, 0016373, 0016374, 0016375, 0016376, 0016377, 0016378, 001637B, 001U074, 001U076, 001U077, 001U079, 001U374, 001U376, 001U377, 001U379, 00B70ZZ, 00B73ZZ, 00B74ZZ, 00T70ZZ, 00T73ZZ, 00T74ZZ |
|  | Occlusion of cerebral arteries | I63.30, I63.50 |
|  | Muscular dystrophies and myopathies | G71, G72 |
|  | Movement diseases | G10, G20, G21.0, G21.11, G21.19, G21.8, G23.0-G23.2, G23.8, G24.02, G24.8, G25.3-G25.5, G25.81-G25.83, G25.89, G25.9, G80.3 |
|  | Devices | T85.09XA, T85.190A, T85.192A, T85.199A, T85.79XA, Z98.2, Z45.41, Z45.42, 00160J0, 00160J1, 00160J2, 00160J3, 00160J4, 00160J5, 00160J6, 00160J7, 00160J8, 00160JB, 00160K0, 00160K1, 00160K2, 00160K3, 00160K4, 00160K5, 00160K6, 00160K7, 00160K8, 00160KB, 00163J0, 00163J1, 00163J2, 00163J3, 00163J4, 00163J5, 00163J6, 00163J7, 00163J8, 00163JB, 00163K0, 00163K1, 00163K2, 00163K3, 00163K4, 00163K5, 00163K6, 00163K7, 00163K8, 00163KB, 001U0J4, 001U0J6, 001U0J7, 001U0J9, 001U0K4, 001U0K6, 001U0K7, 001U0K9, 001U3J4, 001U3J6, 001U3J7, 001U3J9, 001U3K4, 001U3K6, 001U3K7, 001U3K9, 009600Z, 009630Z, 009640Z, 00H00MZ, 00H03MZ, 00H04MZ, 00H60MZ, 00H63MZ, 00H64MZ, 00HE0MZ, 00HE3MZ, 00HE4MZ, 00HU0MZ, 00HU3MZ, 00HU4MZ, 00HV0MZ, 00HV3MZ, 00HV4MZ, 00W60JZ, 00W63JZ, 00W64JZ, 00WU0JZ, 00WU3JZ, 00WU4JZ, 01HY0MZ, 01HY3MZ, 01HY4MZ, 0DH60MZ, 0DH63MZ, 0DH64MZ, 0W110J9, 0W110JB, 0W110JG, 0W110JJ, 3E1Q38X, 3E1Q38Z |
|  | Transplantation | N/A |
| **Cardiovascular** | Heart and great vessel malformations | Q20, Q21.2-Q24, Q25.1-Q26, Q28.2, Q28.3, Q28.9, 02170ZP, 02170ZQ, 02170ZR, 02BK0ZZ, 02LR0ZT, 02LS0ZZ, 02LT0ZZ, 02NH0ZZ, 02RK0JZ, 02RL0JZ, 02RM0JZ, 02RP0JZ, 02RQ07Z, 02RQ0JZ, 02RR07Z, 02RR0JZ, 02SP0ZZ, 02SW0ZZ, 02U70JZ, 02UA0JZ, 02UA3JZ, 02UA4JZ, 02VR0ZT, 02WA0JZ |
|  | Endocardium diseases | I34.0, I34.8, I36.0, I36.8, I37.0, I37.8 |
|  | Cardiomyopathies | I42, I43, I51.5 |
|  | Conduction disorder | I44, I45, I47, I48, I49.0 |
|  | Dysrhythmias | I49.1-I49.5, I49.8, I49.9, R00.1 |
|  | Other | I27.0, I27.1, I27.2, I27.81, I27.89, I27.9, I50.9, I51.7, I51.81, I63.139, I63.239, Z95.1 |
|  | Devices | T82.519A, T82.529A, T82.539A, T82.599A, T82.110A, T82.111A, T82.120A, T82.121A, T82.190A, T82.191A, T82.01XA, T82.02XA, T82.03XA, T82.09XA, T82.211A, T82.212A, T82.213A, T82.218A, T82.221A, T82.222A, T82.223A, T82.228A, T82.518A, T82.528A, T82.538A, T82.598A, T82.6XXA, T82.7XXA, Z95.0, Z95.2, Z95.3, Z95.810-Z95.812, Z95.818, Z45.010, Z45.018, Z45.02, Z45.09, Z95.9, 02H40JZ, 02H40KZ, 02H43JZ, 02H44JZ, 02H44KZ, 02H60JZ, 02H60KZ, 02H63JZ, 02H63KZ, 02H63MZ, 02H64JZ, 02H64KZ, 02H70KZ, 02H73JZ, 02H73KZ, 02H73MZ, 02H74KZ, 02HA0QZ, 02HA0RS, 02HA0RZ, 02HA3QZ, 02HA3RS, 02HA4QZ, 02HA4RS, 02HK0JZ, 02HK0KZ, 02HK3JZ, 02HK3KZ, 02HK3MZ, 02HK4JZ, 02HK4KZ, 02HL0JZ, 02HL0KZ, 02HL0MZ, 02HL3JZ, 02HL3KZ, 02HL3MZ, 02HL4JZ, 02HL4KZ, 02HL4MZ, 02HN0JZ, 02HN0KZ, 02HN0MZ, 02HN3JZ, 02HN3KZ, 02HN3MZ, 02HN4JZ, 02HN4KZ, 02HN4MZ, 02WA0QZ, 02WA0RZ, 02WA3QZ, 02WA3RZ, 02WA4QZ, 02WA4RZ, 03HK0MZ, 03HK3MZ, 03HK4MZ, 03HL0MZ, 03HL3MZ, 03HL4MZ, 03WY0MZ, 03WY3MZ, 03WY4MZ, 0JH600Z, 0JH605Z, 0JH606Z, 0JH607Z, 0JH608Z, 0JH609Z, 0JH60AZ, 0JH60MZ, 0JH60PZ, 0JH630Z, 0JH635Z, 0JH636Z, 0JH637Z, 0JH638Z, 0JH639Z, 0JH63AZ, 0JH63MZ, 0JH63PZ, 0JH70MZ, 0JH73MZ, 0JH800Z, 0JH805Z, 0JH806Z, 0JH807Z, 0JH808Z, 0JH809Z, 0JH80AZ, 0JH80MZ, 0JH80PZ, 0JH830Z, 0JH835Z, 0JH836Z, 0JH837Z, 0JH838Z, 0JH839Z, 0JH83AZ, 0JH83MZ, 0JH83PZ, 0JWT0MZ, 0JWT0PZ, 0JWT3MZ, 0JWT3PZ, 0JWTXMZ, 4B02XSZ, 4B02XTZ, 5A02110, 5A02116, 5A0211D, 5A02210, 5A02216, 5A0221D |
|  | Transplantation | T86.20-T86.22, Z94.1, 02YA0Z0, 02YA0Z1, 02YA0Z2 |
| **Respiratory** | Respiratory malformations | Q30-Q34, P280 |
|  | Chronic respiratory diseases | G47.35, I27.82, I43, J84.112, J96.20, Z90.2 |
|  | Cystic fibrosis | E84 |
|  | Other | 0B110Z4, 0B113Z4, 0B114Z4, 0BTC0ZZ, 0BTC4ZZ, 0BTD0ZZ, 0BTD4ZZ, 0BTF0ZZ, 0BTF4ZZ, 0BTG0ZZ, 0BTG4ZZ, 0BTJ0ZZ, 0BTJ4ZZ, 0BTK0ZZ, 0BTK4ZZ, 0BTL0ZZ, 0BTL4ZZ, 0BTM0ZZ, 0BTM4ZZ, 0CTS0ZZ, 0CTS4ZZ, 0CTS7ZZ, 0CTS8ZZ |
|  | Devices | J95.00-J95.04, J95.09, Z43.0, Z93.0, Z99.0, J95.850, Z99.11, Z99.12, 0B110F4, 0B113F4, 0B114F4, 0B21XFZ, 0BHR0MZ, 0BHR3MZ, 0BHR4MZ, 0BHS0MZ, 0BHS3MZ, 0BHS4MZ, 0BW10FZ, 0BW13FZ, 0BW14FZ, 0JH604Z, 0JH634Z, 0JH804Z, 0JH834Z, 0WQ6XZ2, 3E1F78Z |
|  | Transplantation | T86.810, T86.811, T86.819, Z94.2, 0BYC0Z0, 0BYC0Z1, 0BYC0Z2, 0BYD0Z0, 0BYD0Z1, 0BYD0Z2, 0BYF0Z0, 0BYF0Z1, 0BYF0Z2, 0BYG0Z0, 0BYG0Z1, 0BYG0Z2, 0BYH0Z0, 0BYH0Z1, 0BYH0Z2, 0BYJ0Z0, 0BYJ0Z1, 0BYJ0Z2, 0BYK0Z0, 0BYK0Z1, 0BYK0Z2, 0BYL0Z0, 0BYL0Z1, 0BYL0Z2, 0BYM0Z0, 0BYM0Z1, 0BYM0Z2 |
| **Renal and Urologic** | Congenital anomalies | Q60-Q64 |
|  | Chronic renal failure | N18 |
|  | Other | Z90.5, Z90.6, 0T160Z8, 0T160ZA, 0T164Z8, 0T164ZA, 0T170Z8, 0T170ZA, 0T174Z8, 0T174ZA, 0T180Z8, 0T180ZA, 0T184Z8, 0T184ZA, 0TB60ZZ, 0TB63ZZ, 0TB64ZZ, 0TB67ZZ, 0TB68ZZ, 0TB70ZZ, 0TB73ZZ, 0TB74ZZ, 0TB77ZZ, 0TB78ZZ, 0TT00ZZ, 0TT04ZZ, 0TT10ZZ, 0TT14ZZ, 0TT20ZZ, 0TT24ZZ, 0TT60ZZ, 0TT64ZZ, 0TT67ZZ, 0TT68ZZ, 0TT70ZZ, 0TT74ZZ, 0TT77ZZ, 0TT78ZZ, 0TTB0ZZ, 0TTB4ZZ, 0TTB7ZZ, 0TTB8ZZ, 0TTD0ZZ, 0TTD4ZZ, 0TTD7ZZ, 0TTD8ZZ |
|  | Chronic bladder diseases | G83.4, N31.2, N31.9 |
|  | Devices | T85.71XA, Z93.50-Z93.52, Z93.59, Z93.6, Z91.15, Z99.2, Z43.5, Z43.6, Z46.6, 031209D, 031209F, 03120AD, 03120AF, 03120JD, 03120JF, 03120KD, 03120KF, 03120ZD, 03120ZF, 031309D, 031309F, 03130AD, 03130AF, 03130JD, 03130JF, 03130KD, 03130KF, 03130ZD, 03130ZF, 031409D, 031409F, 03140AD, 03140AF, 03140JD, 03140JF, 03140KD, 03140KF, 03140ZD, 03140ZF, 031509D, 031509F, 03150AD, 03150AF, 03150JD, 03150JF, 03150KD, 03150KF, 03150ZD, 03150ZF, 031609D, 031609F, 03160AD, 03160AF, 03160JD, 03160JF, 03160KD, 03160KF, 03160ZD, 03160ZF, 031709D, 031709F, 03170AD, 03170AF, 03170JD, 03170JF, 03170KD, 03170KF, 03170ZD, 03170ZF, 031809D, 031809F, 03180AD, 03180AF, 03180JD, 03180JF, 03180KD, 03180KF, 03180ZD, 03180ZF, 031909F, 03190AF, 03190JF, 03190KF, 03190ZF, 031A09F, 031A0AF, 031A0JF, 031A0KF, 031A0ZF, 031B09F, 031B0AF, 031B0JF, 031B0KF, 031B0ZF, 031C09F, 031C0AF, 031C0JF, 031C0KF, 031C0ZF, 03WY0JZ, 03WY3JZ, 03WY4JZ, 03WYXJZ, 05HY33Z, 06HY33Z, 0JH60WZ, 0JH60XZ, 0JH63WZ, 0JH63XZ, 0JH80WZ, 0JH80XZ, 0JH83WZ, 0JH83XZ, 0JHD0WZ, 0JHD0XZ, 0JHD3WZ, 0JHD3XZ, 0JHF0WZ, 0JHF0XZ, 0JHF3WZ, 0JHF3XZ, 0JHL0WZ, 0JHL0XZ, 0JHL3WZ, 0JHL3XZ, 0JHM0WZ, 0JHM0XZ, 0JHM3WZ, 0JHM3XZ, 0T130ZB, 0T134ZB, 0T140ZB, 0T144ZB, 0T16079, 0T1607C, 0T1607D, 0T160J9, 0T160JC, 0T160JD, 0T160K9, 0T160KC, 0T160KD, 0T160Z9, 0T160ZC, 0T160ZD, 0T163JD, 0T16479, 0T1647C, 0T1647D, 0T164J9, 0T164JC, 0T164JD, 0T164K9, 0T164KC, 0T164KD, 0T164Z9, 0T164ZC, 0T164ZD, 0T17079, 0T1707C, 0T1707D, 0T170J9, 0T170JC, 0T170JD, 0T170K9, 0T170KC, 0T170KD, 0T170Z9, 0T170ZC, 0T170ZD, 0T173JD, 0T17479, 0T1747C, 0T1747D, 0T174J9, 0T174JC, 0T174JD, 0T174K9, 0T174KC, 0T174KD, 0T174Z9, 0T174ZC, 0T174ZD, 0T18079, 0T1807C, 0T1807D, 0T180J9, 0T180JC, 0T180JD, 0T180K9, 0T180KC, 0T180KD, 0T180Z9, 0T180ZC, 0T180ZD, 0T183JD, 0T18479, 0T1847C, 0T1847D, 0T184J9, 0T184JC, 0T184JD, 0T184K9, 0T184KC, 0T184KD, 0T184Z9, 0T184ZC, 0T184ZD, 0T1B0ZD, 0T1B4ZD, 0T25X0Z, 0T29X0Z, 0T29XYZ, 0T2BX0Z, 0T9000Z, 0T9030Z, 0T9040Z, 0T9070Z, 0T9080Z, 0T9100Z, 0T9130Z, 0T9140Z, 0T9170Z, 0T9180Z, 0T9370Z, 0T9380Z, 0T9470Z, 0T9480Z, 0TQ67ZZ, 0TQ77ZZ, 3E1K38Z, 3E1M39Z, 5A1D60Z |
|  | Transplantation | T86.10-T86.12, Z94.0, 0TY00Z0, 0TY00Z1, 0TY00Z2, 0TY10Z0, 0TY10Z1, 0TY10Z2 |
| **Gastrointestinal** | Congenital anomalies | Q39.0-Q39.4, Q41-Q45 |
|  | Chronic liver disease and cirrhosis | K73, K74, K75.4, K760-K763, K765, K768 |
|  | Inflammatory bowel diseases | K50, K51 |
|  | Other | I82.0, K55.1, K56.2, K59.3, Z98.0, Z90.3, Z90.49, 0CT70ZZ, 0CT7XZZ, 0D13079, 0D1307A, 0D1307B, 0D1607A, 0D160ZA, 0DT50ZZ, 0DT54ZZ, 0DT57ZZ, 0DT58ZZ, 0DT60ZZ, 0DT64ZZ, 0DT67ZZ, 0DT68ZZ, 0DT80ZZ, 0DT84ZZ, 0DT87ZZ, 0DT88ZZ, 0DT90ZZ, 0DT94ZZ, 0DT97ZZ, 0DT98ZZ, 0DTE0ZZ, 0DTE4ZZ, 0DTE7ZZ, 0DTE8ZZ, 0FT00ZZ, 0FT04ZZ, 0FTG0ZZ, 0FTG4ZZ |
|  | Devices | K94.20, K94.22, K94.23, K94.29, Z93.1-Z93.4, Z43.1-Z43.4, Z46.51, Z46.59, 0D11074, 0D110J4, 0D110K4, 0D110Z4, 0D113J4, 0D11474, 0D114J4, 0D114K4, 0D114Z4, 0D15074, 0D150J4, 0D150K4, 0D150Z4, 0D153J4, 0D15474, 0D154J4, 0D154K4, 0D154Z4, 0D16074, 0D160J4, 0D160J9, 0D160JA, 0D160K4, 0D160K9, 0D160KA, 0D160Z4, 0D163J4, 0D16474, 0D164J4, 0D164J9, 0D164JA, 0D164K4, 0D164K9, 0D164KA, 0D164Z4, 0D16874, 0D168J4, 0D168J9, 0D168JA, 0D168K4, 0D168K9, 0D168KA, 0D168Z4, 0D1B0Z4, 0D1B4Z4, 0D1B8Z4, 0D1H0Z4, 0D1H4Z4, 0D1H8Z4, 0D1K0Z4, 0D1K4Z4, 0D1K8Z4, 0D1L0Z4, 0D1L4Z4, 0D1L8Z4, 0D1N0Z4, 0D1N4Z4, 0D1N8Z4, 0D20X0Z, 0D20XUZ, 0D20XYZ, 0D787ZZ, 0D7E7ZZ, 0DBB7ZZ, 0DH50DZ, 0DH50UZ, 0DH53DZ, 0DH53UZ, 0DH54DZ, 0DH54UZ, 0DH57DZ, 0DH57UZ, 0DH58DZ, 0DH58UZ, 0DH63UZ, 0DH64UZ, 0DHA3UZ, 0DHA4UZ, 0DHA8UZ, 0DN87ZZ, 0DNE7ZZ, 0DW04UZ, 0DW08UZ, 0WQFXZ2, 3E1G78Z, 3E1H78Z |
|  | Transplantation | T86.40-T86.42, T86.890, T86.891, T86.899, T86.850, T86.851, T86.859, Z94.4, Z94.82, Z94.83, 0DY80Z0, 0DY80Z1, 0DY80Z2, 0DYE0Z0, 0DYE0Z1, 0DYE0Z2, 0FY00Z0, 0FY00Z1, 0FY00Z2, 0FYG0Z0, 0FYG0Z1, 0FYG0Z2, 3E030U0, 3E030U1, 3E033U0, 3E033U1, 3E0J3U0, 3E0J3U1, 3E0J7U0, 3E0J7U1, 3E0J8U0, 3E0J8U1 |
| **Hematologic or Immunologic** | Hereditary anemias | D55-D58 |
|  | Aplastic anemias | D60-D61, D71 |
|  | Hereditary immunodeficiency | D80-D89, D72.0, M30.3, M35.9 |
|  | Coagulation/hemorrhagic | D66, D68.2, D69.41, D69.42, D69.49 |
|  | Leukopenia | D70.0, D70.4 |
|  | Hemophagocytic Syndromes | D76.1-D76.3 |
|  | Sarcoidosis | D86.9 |
|  | Acquired immunodeficiency | B20-B24 |
|  | Polyarteritis nodosa and related conditions | M30.0, M31.0, M31.1, M31.30, M31.4, M31.6 |
|  | Diffuse diseases of connective tissue | M32.10, M33.90, M34.0, M34.1, M34.9 |
|  | Other | 07TP0ZZ, 07TP4ZZ |
|  | Devices | N/A |
|  | Transplantation | 07YP0Z0, 07YP0Z1, 07YP0Z2, 30230AZ, 30230G0, 30230G1, 30230X0, 30230X1, 30230Y0, 30230Y1, 30233AZ, 30233G0, 30233G1, 30233X0, 30233X1, 30233Y0, 30233Y1, 30240AZ, 30240G0, 30240G1, 30240X0, 30240X1, 30240Y0, 30240Y1, 30243AZ, 30243G0, 30243G1, 30243X0, 30243X1, 30243Y0, 30243Y1, 30250G0, 30250G1, 30250X0, 30250X1, 30250Y0, 30250Y1, 30253G0, 30253G1, 30253X0, 30253X1, 30253Y0, 30253Y1, 30260G0, 30260G1, 30260X0, 30260X1, 30260Y0, 30260Y1, 30263G0, 30263G1, 30263X0, 30263X1, 30263Y0, 30263Y1 |
| **Metabolic** | Amino acid metabolism | E70.0, E70.2, E70.3, E70.4, E70.8, E71.0-E71.5, E72.0-E72.4, E72.8, E72.9 |
|  | Carbohydrate metabolism | E74.0-E74.4, E74.8, E74.9 |
|  | Lipid metabolism | E75, E77.0, E77.1, E78.0-E78.4, E78.5-E78.9, E88.1, E88.8 |
|  | Storage disorder | E76.0-E76.3, E85 |
|  | Other metabolic disorders | 277.4, E79.1, E79.8, E80.4-E80.7, E83.0, E83.1, E83.3, E83.4, D84.1, E88, H49.8 |
|  | Endocrine disorders | E00.9, E23.0, E23.2, E22.2, E23.3, E23.7, E24.0, E24.2, E24.3, E24.8, E24.9, E26.81, E25.0, E25.8, E25.9, 0GT00ZZ, 0GT04ZZ, 0GT40ZZ, 0GT44ZZ, 0GTK0ZZ, 0GTK4ZZ, 0GTR0ZZ, 0GTR4ZZ, 0UT20ZZ, 0UT24ZZ, 0UT27ZZ, 0UT28ZZ, 0UT2FZZ, 0UT40ZZ, 0UT44ZZ, 0UT47ZZ, 0UT48ZZ, 0UT70ZZ, 0UT74ZZ, 0UT90ZZ, 0UT94ZZ, 0UT97ZZ, 0UT98ZZ, 0UT9FZZ, 0UTC0ZZ, 0UTC7ZZ, 0UTC8ZZ, 0VTC0ZZ, 0VTC4ZZ, 0W4M070, 0W4M0J0, 0W4M0K0, 0W4M0Z0, 0W4N071, 0W4N0J1, 0W4N0K1, 0W4N0Z1 |
|  | Devices | Z46.81, Z96.41, 0JH60VZ, 0JH63VZ, 0JH70VZ, 0JH73VZ, 0JH80VZ, 0JH83VZ, 0JHD0VZ, 0JHD3VZ, 0JHF0VZ, 0JHF3VZ, 0JHG0VZ, 0JHG3VZ, 0JHH0VZ, 0JHH3VZ, 0JHL0VZ, 0JHL3VZ, 0JHM0VZ, 0JHM3VZ, 0JHN0VZ, 0JHN3VZ, 0JHP0VZ, 0JHP3VZ, 0JHT0VZ, 0JHT3VZ |
|  | Transplantation | N/A |
| **Other Congenital or Genetic Defect** | Chromosomal anomalies | Q90.9, Q91.3, Q91.4, Q91.7, Q92.8, Q93, Q95.0, Q96.9, Q97, Q98, Q99.8, Q99.9 |
|  | Bone and join anomalies | E34.3, M41.0, M41.2, M41.30, M41.8, M41.9, M43.30, M96.5, Q72.2, Q75.0, Q75.2, Q75.9, Q76.0-Q76.2, Q76.4-Q76.7, Q77, Q78.0-Q78.4, Q78.8, Q78.9 |
|  | Diaphragm and abdominal wall | K44.9, Q79.0-Q79.5, Q79.9, Q79.59 |
|  | Other congenital anomalies | Q81, Q87.1-Q87.3, Q87.40, Q87.81, Q87.89, Q89.7, Q89.9, Q99.2 |
| **Malignancy** | Neoplasms | C00-C96, D01-D09, D3A.0, D37-D49, Q85.0, 3E00X05, 3E01305, 3E02305, 3E03005, 3E03305, 3E04005, 3E04305, 3E05005, 3E05305, 3E06005, 3E06305, 3E0A305, 3E0F305, 3E0F705, 3E0F805, 3E0G305, 3E0G705, 3E0G805, 3E0H305, 3E0H705, 3E0H805, 3E0J305, 3E0J705, 3E0J805, 3E0K305, 3E0K705, 3E0K805, 3E0L305, 3E0L705, 3E0M305, 3E0M705, 3E0N305, 3E0N705, 3E0N805, 3E0P305, 3E0P705, 3E0P805, 3E0Q305, 3E0Q705, 3E0R305, 3E0S305, 3E0V305, 3E0W305, 3E0Y305, 3E0Y705 |
|  | Devices | N/A |
|  | Transplantation | T86.00-T86.02, T86.09, Z94.81, Z94.84 |
| **Premature and Neonatal** | Fetal malnutrition | P05.01, P05.11, P05.02, P05.12, P05.2, P05.9 |
|  | Extreme immaturity | P07.01, P07.02, P07.21-P07.25 |
|  | Cerebral hemorrhage at birth | P10.0, P10.1, P10.4, P52.4, P52.8 |
|  | Spinal cord injury at birth | P11.5 |
|  | Birth asphyxia | P21.0, P21.9, P84 |
|  | Respiratory diseases | P25.0-P25.3, P25.8, P27.0, P27.1, P27.8 |
|  | Hypoxic-ischemic encephalopathy | P91.6 |
|  | Other | P35.0, P35.1, P25.21, P25.22, P56.0, P57.0, P57.8, P61.3, P61.4, P77.3, P83.2, P91.2 |
| **Miscellaneous, Not Elsewhere Classified** | Devices | T84.019A, T84.029A, T84.039A, T84.049A, T84.059A, T84.069A, T84.099A, T84.498A, T84.119A, T84.129A, T84.199A, T84.498A, T84.50XA, T84.60XA, , T84.7XXA, T86.90-T86.92, T86.99, T86.10-T86.12, T86.40-T86.42, T86.20-T86.22, T86.810, T86.811, T86.819, T86.00-T86.02, T86.09, T86.890, T86.891, T86.899, T86.850, T86.851, T86.859, T86.5, T86.890, T86.891, T86.899, T87.0X9, T87.1X9, T87.2, Y83.1, Y83.3, Z99.81, 0RG00J0, 0RG00J1, 0RG00JJ, 0RG00K0, 0RG00K1, 0RG00KJ, 0RG00Z0, 0RG00Z1, 0RG00ZJ, 0RG03J0, 0RG03J1, 0RG03JJ, 0RG03K0, 0RG03K1, 0RG03KJ, 0RG03Z0, 0RG03Z1, 0RG03ZJ, 0RG04J0, 0RG04J1, 0RG04JJ, 0RG04K0, 0RG04K1, 0RG04KJ, 0RG04Z0, 0RG04Z1, 0RG04ZJ, 0RG10J0, 0RG10J1, 0RG10JJ, 0RG10K0, 0RG10K1, 0RG10KJ, 0RG10Z0, 0RG10Z1, 0RG10ZJ, 0RG13J0, 0RG13J1, 0RG13JJ, 0RG13K0, 0RG13K1, 0RG13KJ, 0RG13Z0, 0RG13Z1, 0RG13ZJ, 0RG14J0, 0RG14J1, 0RG14JJ, 0RG14K0, 0RG14K1, 0RG14KJ, 0RG14Z0, 0RG14Z1, 0RG14ZJ, 0RG40J0, 0RG40J1, 0RG40JJ, 0RG40K0, 0RG40K1, 0RG40KJ, 0RG40Z0, 0RG40Z1, 0RG40ZJ, 0RG43J0, 0RG43J1, 0RG43JJ, 0RG43K0, 0RG43K1, 0RG43KJ, 0RG43Z0, 0RG43Z1, 0RG43ZJ, 0RG44J0, 0RG44J1, 0RG44JJ, 0RG44K0, 0RG44K1, 0RG44KJ, 0RG44Z0, 0RG44Z1, 0RG44ZJ, 0RG60J0, 0RG60J1, 0RG60JJ, 0RG60K0, 0RG60K1, 0RG60KJ, 0RG60Z0, 0RG60Z1, 0RG60ZJ, 0RG63J0, 0RG63J1, 0RG63JJ, 0RG63K0, 0RG63K1, 0RG63KJ, 0RG63Z0, 0RG63Z1, 0RG63ZJ, 0RG64J0, 0RG64J1, 0RG64JJ, 0RG64K0, 0RG64K1, 0RG64KJ, 0RG64Z0, 0RG64Z1, 0RG64ZJ, 0RGA0J0, 0RGA0J1, 0RGA0JJ, 0RGA0K0, 0RGA0K1, 0RGA0KJ, 0RGA0Z0, 0RGA0Z1, 0RGA0ZJ, 0RGA3J0, 0RGA3J1, 0RGA3JJ, 0RGA3K0, 0RGA3K1, 0RGA3KJ, 0RGA3Z0, 0RGA3Z1, 0RGA3ZJ, 0RGA4J0, 0RGA4J1, 0RGA4JJ, 0RGA4K0, 0RGA4K1, 0RGA4KJ, 0RGA4Z0, 0RGA4Z1, 0RGA4ZJ, 0SG00J0, 0SG00J1, 0SG00JJ, 0SG00K0, 0SG00K1, 0SG00KJ, 0SG00Z0, 0SG00Z1, 0SG00ZJ, 0SG03J0, 0SG03J1, 0SG03JJ, 0SG03K0, 0SG03K1, 0SG03KJ, 0SG03Z0, 0SG03Z1, 0SG03ZJ, 0SG04J0, 0SG04J1, 0SG04JJ, 0SG04K0, 0SG04K1, 0SG04KJ, 0SG04Z0, 0SG04Z1, 0SG04ZJ |
|  | Transplantation | T86.5, T86.90-T86.92, T86.99, T86.890, T86.891, T86.899 |
